# Update: Influenza Activity — United States and Worldwide, May 19–September 28, 2013

**Published:** 2013-10-25

**Authors:** World Health Organization Collaborating Center for Surveillance, Epidemiology, and Control of Influenza, Scott Epperson, Lynnette Brammer, Lenee Blanton, Desiree Mustaquim, Krista Kniss, Craig Steffens, Anwar Isa Abd Elal, Larisa Gubareva, Teresa Wallis, Jackie Katz, Julie Villanueva, Xiyan Xu, Joseph Bresee, Nancy Cox, Lyn Finelli, Ikwo Oboho

**Affiliations:** Influenza Div, National Center for Immunization and Respiratory Diseases; EIS Officer, CDC

During May 19–September 28, 2013,* the United States experienced low levels of seasonal influenza activity overall. Influenza A (H1N1) pdm09 (pH1N1), influenza A (H3N2), and influenza B viruses were detected worldwide and were identified sporadically in the United States. In June, influenza A (H3N2) variant† viruses (H3N2)v were first detected in Indiana, and between June 18 and September 28, a total of 20 cases of influenza A variant viruses ([H3N2]v and influenza A (H1N1) variant [H1N1]v) were reported from five states. This report summarizes influenza activity in the United States and worldwide from May 19 through September 28, 2013.

## United States

The U.S. influenza surveillance system is a collaborative effort between CDC and federal, state, local, and territorial partners. CDC uses eight data sources to collect influenza information (1), six of which operate year-round: 1) U.S. World Health Organization (WHO) collaborating laboratories, 2) the National Respiratory and Enteric Virus Surveillance System (NREVSS), 3) reports of human infections with novel influenza A viruses, 4) the U.S. Outpatient Influenza-like Illness Surveillance Network (ILINet), 5) the 122 Cities Mortality Reporting System, and 6) the Influenza-Associated Pediatric Mortality Reporting System.§

During May 19–September 28, WHO and NREVSS collaborating laboratories in the United States tested 52,150 respiratory specimens for influenza; 2,013 (3.9%) tested positive for influenza (Figure). During summers of the previous 6 years (excluding the summer during the 2009 pandemic) the average number of respiratory specimens tested for influenza was 33,780 (range: 22,961–51,734), with an average of 835 (2.5%) specimens testing positive (range: 175–3,160).

Of the 2,013 specimens positive for influenza in the summer months of 2013, 1,403 (70%) were influenza A viruses, and 610 (30%) were influenza B viruses. Influenza B viruses were reported more frequently than influenza A viruses from late May until early June, and influenza A (pH1N1) viruses were more commonly reported than influenza A (H3N2) viruses from mid-June to September. Of the 1,403 influenza A viruses, 621 (44%) were subtyped: 402 (65%) were influenza A (pH1N1) viruses, 201 (32%) were influenza A (H3N2) viruses, and 18 (3%) were influenza A (H3N2)v viruses. Influenza viruses were reported from the District of Columbia, Puerto Rico, and 45 states in all 10 U.S. Department of Health and Human Services regions.¶ The largest number of influenza-positive specimens (971) came from the southeastern United States (Region 4), followed by the western states (Region 9), with 505 influenza-positive specimens.

During May 19–September 28 data from ILINet indicated that the weekly percentage of outpatient visits to ILINet providers for influenza-like illness (ILI)** remained below the national baseline of 2.2% (range: 0.7%–1.2%).†† The percentage of deaths attributed to pneumonia and influenza (P&I), as reported by the 122 Cities Mortality Reporting System, remained below the epidemic threshold (range: 5.2% to 6.5%).§§ Two influenza-associated pediatric deaths occurring during May 19–September 28 were reported; one was associated with an influenza A (pH1N1) virus and one was associated with an influenza A virus for which subtyping was not performed.

## Novel Influenza A Virus Infection

Between May 19 and September 28, a total of 20 cases of influenza A variant viruses (18 [H3N2]v and two [H1N1]v) were reported from five states (Arkansas [two], Illinois [one], Indiana [14], Michigan [two] and Ohio [one]). The 20 cases reported resulted in one influenza A (H3N2)v–associated hospitalization and no deaths. Although cases have been identified from five states, Indiana reported 14 (70%) of the 20 cases. In all 20 cases, contact with swine in the week before illness onset was reported. No ongoing community transmission of these viruses has been detected. The median age of patients was 6.5 years (range: 2–69 years); 65% were female (Influenza Division, National Center for Immunization and Respiratory Diseases, CDC, unpublished data, 2013).

## Worldwide

During May 19–September 28, typical seasonal patterns of influenza activity occurred in the temperate climate Southern Hemisphere countries. In Australia and New Zealand, influenza activity began in early August and decreased in mid-September. Influenza A viruses predominated in both countries with influenza A (H3N2) viruses identified more frequently than influenza A (pH1N1) viruses. Influenza B viruses were also identified in both countries. In South Africa, after a peak in influenza activity caused by influenza A (pH1N1) in June, a second, smaller peak was observed in early August because of increased influenza A (H3N2) and influenza B virus circulation. In temperate areas of South America, influenza activity peaked in June and declined through September. Influenza A viruses were reported more frequently than influenza B viruses, and influenza A (pH1N1) was the predominant virus reported by Argentina, Chile, and Uruguay. Influenza A (H3N2) viruses predominated in Paraguay (2).

Influenza activity was reported from countries with tropical influenza seasonality. The overall level of activity compared with previous seasons, and the predominant subtype varied by country. In the Caribbean and Central America, influenza activity peaked in early July and declined during August and September, with cocirculation of influenza A (pH1N1) and influenza A (H3N2) viruses. In tropical South America, influenza A (pH1N1) viruses predominated, with two peaks of activity: the first in June, primarily the result of activity in Brazil and Columbia, and a second peak in late July, the result of increased activity in Ecuador and Peru. South Asia and Southeast Asia saw a decrease in influenza activity during September. Different combinations of types and subtypes of influenza cocirculated in several countries, including Cambodia, India, China, Vietnam, and Thailand. In temperate climate countries in North America and Europe, influenza activity remained low, with small numbers of influenza A (H3N2), influenza A (pH1N1), and influenza B viruses identified. During February 19–September 28, a total of 135 cases of human infection with avian influenza A (H7N9) virus were reported (3). No human cases of avian influenza A (H7N9) virus infection have been detected outside of China (3).

## Antigenic Characterization of Influenza Virus Isolates

The recommended components for the 2013–14 influenza trivalent influenza vaccines are an A/California/7/2009 (H1N1)-like virus, an influenza A (H3N2) virus antigenically like the cell-propagated prototype virus A/Victoria/361/2011 (e.g., A/Texas/50/2012), and a B/Massachusetts/2/2012-like (B/Yamagata lineage) virus (4). For quadrivalent vaccines, a B/Brisbane/60/2008-like (B/Victoria lineage) virus is recommended (4).

CDC antigenically characterized 342 influenza viruses collected during May 19–September 28 from laboratories worldwide, including 227 influenza A (pH1N1) viruses, 85 influenza A (H3N2) viruses, and 30 influenza B viruses. A subset of these viruses was genetically characterized. Most viruses tested were propagated in mammalian cell cultures; isolation rates of current human influenza viruses are higher in mammalian cell cultures than in eggs. However, egg-propagated (EP) vaccine viruses are used widely for production of influenza vaccines because most influenza virus vaccines are egg-based. Propagation of influenza viruses in eggs might lead to isolation of viruses that differ antigenically and genetically from viruses from corresponding clinical samples isolated in mammalian cell cultures. In addition, mammalian cell-propagated (CP) viruses are genetically more representative of viruses present in original clinical specimens (5,6). Therefore, it is important to select EP vaccine viruses that are antigenically and genetically most similar to their CP counterparts.

All but two (99%) influenza A (pH1N1) viruses analyzed (94 from North America, 131 from South America, one from Asia, and one from Oceania) were antigenically similar to EP A/California/7/2009, the influenza A (pH1N1) vaccine component. Most EP and CP influenza A (pH1N1) viruses analyzed during this time had similar antigenic properties. All 85 influenza A (H3N2) viruses (40 from the United States, 40 from South America, two from Oceania, and three from Asia) were antigenically similar to many current CP reference viruses, including A/Victoria/361/2011 and A/Texas/50/2012. The majority (60%) of CP influenza A (H3N2) viruses collected and characterized since February 2013 were antigenically similar to the EP A/Texas/50/2012 vaccine virus, while less than 10% of these viruses were antigenically similar to EP A/Victoria/361/2011. For this reason, WHO recommended that the EP A/Texas/50/2012 vaccine virus replace EP A/Victoria/361/2011 as the influenza A (H3N2) vaccine component for the 2013–14 Northern Hemisphere and the 2014 Southern Hemisphere influenza seasons (4).

Of the 30 influenza B viruses collected and analyzed during this period, 13 (43%) belonged to the B/Yamagata lineage (five from the United States, seven from South America, and one from Asia), and were antigenically similar to the B/Massachusetts/2/2012 virus, the influenza B component for the 2013–14 Northern Hemisphere trivalent vaccine. The remaining 17 viruses tested (57%, one from the United States and 16 from South America) belonged to the B/Victoria lineage and were antigenically similar to the B/Brisbane/60/2008 virus, which is the B/Victoria-lineage component of the 2013–14 Northern Hemisphere quadrivalent influenza vaccine. Globally, B/Yamagata lineage viruses predominated during February 1–September 28 (4). Most EP and CP influenza B viruses analyzed during this time had similar antigenic properties. The WHO recommendation for influenza vaccine composition for the 2014 Southern Hemisphere season remained the same as that for the 2013–14 Northern Hemisphere season (4).

## Antiviral Resistance Profiles of Influenza Virus Isolates

The WHO Collaborating Center for Surveillance, Epidemiology, and Control of Influenza at CDC tested 336 specimens collected during May 19–September 28 for resistance to influenza antiviral medications. Of the 336 specimens tested for resistance to the neuraminidase inhibitor medications oseltamivir and zanamivir, 196 were collected internationally (127 influenza A [pH1N1], 42 influenza A [H3N2], and 27 influenza B viruses), and 140 were from U.S. specimens (93 influenza A [pH1N1], 39 influenza A [H3N2], and eight influenza B viruses). Two of the 93 influenza A (pH1N1) viruses from the United States were found to be resistant to oseltamivir and contained the H275Y substitution in the neuraminidase gene; all the viruses were sensitive to zanamivir. High levels of resistance to the adamantanes (amantadine and rimantadine) persist among influenza A (pH1N1) viruses and influenza A (H3N2) viruses currently circulating globally (7).

What is already known on this topic?

CDC collects, compiles, and analyzes data on influenza activity year-round in the United States. The influenza season generally begins in the fall and continues through the winter and spring months; however, the timing and severity of circulating influenza viruses can vary by geographic location and season.

What is added by this report?

The United States experienced low levels of influenza activity during May 19–September 28, 2013, and influenza A (H1N1) pdm09 (pH1N1), influenza A (H3N2), and influenza B viruses were identified sporadically. Twenty cases of influenza A variant viruses (influenza A [H3N2]v and influenza A [H1N1]v) were detected in five states, all of which were associated with swine contact. The majority of recent seasonal influenza viruses are antigenically similar to the influenza vaccine for the 2013–14 season.

What are the implications for public health practice?

To prevent influenza and its associated complications, influenza vaccination is recommended in all persons aged ≥6 months. Year-round influenza surveillance provides critical information for planning interventions to prevent and control influenza, developing vaccine recommendations and antiviral treatment guidance, and presenting information to the media and public regarding the progress and severity of the influenza season.

### Editorial Note

During May 19–September 28, 2013, influenza A (pH1N1), influenza A (H3N2), and influenza B viruses cocirculated worldwide. In the United States, similar levels of seasonal influenza viruses were detected compared with summer months of previous years (excluding the 2009 pandemic), and influenza A viruses were predominant. Although neither the influenza viruses that will predominate nor the severity of influenza-related disease during the 2013–14 season in the United States can be predicted, antigenic characterization of viral isolates submitted during the summer demonstrated that the majority of influenza viruses were antigenically similar to the influenza vaccine strains contained in the 2013–14 Northern Hemisphere vaccine.

Compared with the summer of 2012, fewer human infections with novel influenza A viruses were identified in the United States in the summer of 2013. Since the first identification of H3N2v viruses in humans, direct contact with swine has been documented in most cases, but limited person-to-person spread is suspected in a small number. Consistent with the age distribution of patients, serologic studies suggest there is little or no existing cross-reactive antibody to H3N2v in young children, but a substantial proportion of adolescents and younger adults have cross-reactive antibodies (8,9). Where community transmission of this virus has not been identified, the potential for this virus to develop the ability to transmit efficiently from person-to-person is of concern. Rapid and intensive investigation of each novel influenza A case remains necessary to evaluate the spread of disease and the possibility of person-to-person transmission. While seasonal influenza viruses are circulating at low levels, state and local health departments should consider increased specimen collection among patients with ILI who 1) seek care at an ILINet provider; 2) are part of an ILI outbreak among children in child-care or school settings (because these settings were associated with person-to-person H3N2v, virus transmission in 2011); 3) have an unusual or severe presentation of ILI, including hospitalized persons; or 4) have medically attended ILI or acute respiratory infection, especially among children in jurisdictions where H3N2v cases have occurred (10).

Annual influenza vaccination remains the best method for preventing influenza and its associated complications (4). For optimal protection against seasonal influenza viruses, annual influenza vaccination is recommended for all persons aged ≥6 months each year, regardless of whether the vaccine virus strains have changed since the previous season. In addition to the types of influenza vaccines available during the last season, several new influenza vaccines have been approved for use and will be available for the 2013–14 season, including a quadrivalent live attenuated influenza vaccine (LAIV4), quadrivalent inactivated influenza vaccines (IIV4), a trivalent cell culture-based inactivated influenza vaccine (ccIIV3), and a new recombinant hemagglutinin vaccine (RIV3) (4). For many vaccine recipients, more than one type or brand of vaccine might be appropriate within indications and Advisory Committee on Immunization Practices (ACIP) recommendations. Where more than one type of vaccine is appropriate and available, ACIP has not recommended any one influenza vaccine product over another. Children aged 6 months–8 years who are being vaccinated for the first time should receive 2 doses of influenza vaccine. For children aged 6 months–8 years who have received influenza vaccination before, health-care providers should consult the ACIP guidelines to assess whether 1 or 2 doses are required (4).

Treatment with influenza antiviral medications is recommended as early as possible for patients with confirmed or suspected influenza (either seasonal influenza or variant influenza virus infection) who have severe, complicated, or progressive illness; who require hospitalization; or who are at higher risk for influenza-related complications (7).¶¶

Influenza surveillance reports for the United States are normally posted online weekly and are available at http://www.cdc.gov/flu/weekly. Additional information regarding influenza viruses, influenza surveillance, influenza vaccines, influenza antiviral medications, and novel influenza A virus infections in humans is available at http://www.cdc.gov/flu.

## Figures and Tables

**FIGURE:**
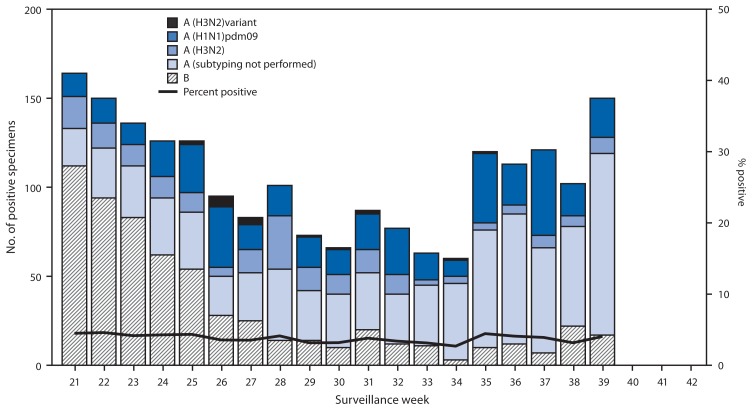
Number* and percentage of respiratory specimens testing positive for influenza reported by World Health Organization and National Respiratory and Enteric Virus Surveillance System collaborating laboratories in the United States, by type, subtype, and week — United States, May 19–September 28, 2013^†^ * N = 2,013. ^†^ As of September 28, 2013.

## Footnotes

* Data as of October 18, 2013.

† Influenza viruses that circulate in swine are called swine influenza viruses when isolated from swine, but are called variant influenza viruses when isolated from humans. A variant virus (human isolate) might have the M gene alone or the M gene plus other genes from the influenza A (pH1N1) virus, along with other genetic changes. Seasonal influenza A (H3N2) viruses that circulate worldwide in the human population have significant antigenic and genetic differences from influenza A (H3N2) viruses circulating in swine. Additional information is available at http://www.who.int/influenza/gisrs_laboratory/terminology_ah3n2v/en/index.html.

§ The CDC influenza surveillance system collects five categories of information from the eight data sources: 1) viral surveillance (World Health Organization collaborating laboratories, the National Respiratory and Enteric Virus Surveillance System, and human infections with novel influenza A viruses); 2) outpatient illness surveillance (U.S. Outpatient Influenza-like Illness Surveillance Network); 3) mortality (122 Cities Mortality Reporting System and influenza-associated pediatric mortality reports); 4) hospitalizations (FluSurv-NET, which includes the Emerging Infections Program and surveillance in five additional states); and 5) summary of the geographic spread of influenza (state and territorial epidemiologist reports).

¶ The 10 regions include the following jurisdictions: *Region 1:* Connecticut, Maine, Massachusetts, New Hampshire, Rhode Island, and Vermont; *Region 2:* New Jersey, New York, Puerto Rico, and the U.S. Virgin Islands; *Region 3:* Delaware, District of Columbia, Maryland, Pennsylvania, Virginia, and West Virginia; *Region 4:* Alabama, Florida, Georgia, Kentucky, Mississippi, North Carolina, South Carolina, and Tennessee; *Region 5:* Illinois, Indiana, Michigan, Minnesota, Ohio, and Wisconsin; *Region 6:* Arkansas, Louisiana, New Mexico, Oklahoma, and Texas; *Region 7:* Iowa, Kansas, Missouri, and Nebraska; *Region 8:* Colorado, Montana, North Dakota, South Dakota, Utah, and Wyoming; *Region 9:* Arizona, California, Hawaii, Nevada, American Samoa, Commonwealth of the Northern Mariana Islands, Federated States of Micronesia, Guam, Marshall Islands, and Republic of Palau; *Region 10:* Alaska, Idaho, Oregon, and Washington.

** Defined as a temperature of ≥100°F (≥37.8°C), oral or equivalent, and cough and/or sore throat, without a known cause other than influenza.

†† The national and regional baselines are the mean percentage of visits for ILI during noninfluenza weeks for the previous three seasons plus two standard deviations. Noninfluenza weeks are defined as periods of 2 or more consecutive weeks in which each week accounted for <2% of the season’s total number of specimens that tested positive for influenza. National and regional percentages of patient visits for ILI are weighted on the basis of state population. Use of the national baseline for regional data is not appropriate.

§§ The seasonal baseline proportion of P&I deaths is projected using a robust regression procedure in which a periodic regression model is applied to the observed percentage of deaths from P&I that were reported by the 122 Cities Mortality Reporting System during the preceding 5 years. The epidemic threshold is set at 1.645 standard deviations above the seasonal baseline.

¶¶ Persons at higher risk include 1) children aged <5 years (especially those aged <2 years); 2) adults aged ≥65 years; 3) persons with chronic pulmonary (including asthma), cardiovascular (except hypertension alone), renal, hepatic, hematologic (including sickle cell disease), metabolic disorders (including diabetes mellitus), or neurologic and neurodevelopment conditions (including disorders of the brain, spinal cord, peripheral nerve, and muscle, such as cerebral palsy, epilepsy [seizure disorders], stroke, intellectual disability [mental retardation], moderate to severe developmental delay, muscular dystrophy, or spinal cord injury); 4) persons with immunosuppression, including that caused by medications or by human immunodeficiency virus infection; 5) women who are pregnant or postpartum (within 2 weeks after delivery); 6) persons aged ≤18 years who are receiving long-term aspirin therapy; 7) American Indians/Alaska Natives; 8) persons who are morbidly obese (i.e., body mass index ≥40); and 9) residents of nursing homes and other chronic-care facilities.
